# A New Dibenzoquinoxalineimide-Based Wide-Bandgap Polymer Donor for Polymer Solar Cells

**DOI:** 10.3390/polym14173590

**Published:** 2022-08-30

**Authors:** Xin Wang, Zongtao Wang, Mingwei Li, Lijun Tu, Ke Wang, Dengping Xiao, Qiang Guo, Ming Zhou, Xianwen Wei, Yongqiang Shi, Erjun Zhou

**Affiliations:** 1 Key Laboratory of Functional Molecular Solids, Ministry of Education, and School of Chemistry and Materials Science, Anhui Normal University, Wuhu 241002, China; 2 School of Materials Science and Engineering, Henan Institute of Advanced Technology, Zhengzhou University, Zhengzhou 450001, China; 3 CAS Center for Excellence in Nanoscience, National Center for Nanoscience and Technology, Beijing 100190, China; 4 State Key Laboratory of Oil and Gas Reservoir Geology and Exploitation, School of New Energy and Materials, Southwest Petroleum University, Chengdu 610500, China

**Keywords:** wide bandgap, donor–acceptor, imide, polymer solar cells

## Abstract

The molecular design of a wide-bandgap polymer donor is critical to achieve high-performance organic photovoltaic devices. Herein, a new dibenzo-fused quinoxalineimide (BPQI) is successfully synthesized as an electron-deficient building block to construct donor–acceptor (D–A)-type polymers, namely P(BPQI-BDT) and P(BPQI-BDTT), using benzodithiophene and its derivative, which bears different side chains, as the copolymerization units. These two polymers are used as a donor, and the narrow bandgap (2,20-((2Z,20Z)-((12,13-bis(2-ethylhexyl)-3,9-diundecyl-12,13-dihydro-[1,2,5]thiadiazolo [3,4-e]thieno[2,″30′:4′,50]thieno[20,30:4,5]pyrrolo[3,2g]thieno[20,30:4,5]thieno[3,2-b]indole-2,10 diyl)bis(methanylylidene))bis(5,6-difluoro-3-oxo-2,3-dihydro-1H-indene-2,1-diylidene))dimalononitrile) Y6 is used as an acceptor to fabricate bulk heterojunction polymer solar cell devices. Y6, as a non-fullerene receptor (NFA), has excellent electrochemical and optical properties, as well as a high efficiency of over 18%. The device, based on P(BPQI-BDTT):Y6, showed power conversion efficiencies (PCEs) of 6.31% with a *J*_SC_ of 17.09 mA cm^−2^, an open-circuit voltage (*V*_OC_) of 0.82 V, and an FF of 44.78%. This study demonstrates that dibenzo-fused quinoxalineimide is a promising building block for developing wide-bandgap polymer donors.

## 1. Introduction

Organic solar cells (OSCs) have attracted a lot of attention from both academia and industry due to their versatile advantages, such as their light weight, stretchability, and solution processability [1,2,3,4,5,6]. Following great efforts in recent years, power conversion efficiencies (PCEs) were achieved in the range of 18–19% [7,8,9]. The remarkable progress in organic photovoltaics (OPVs) mainly depends on the development of new polymer donors and non-fullerene small molecule electron acceptors (NFSMEAs) [10,11,12,13,14,15]. Recently, various narrow-bandgap NFSMEAs have been designed and have achieved excellent performances in OPVs when paired with wide-bandgap polymer donors. Most NFSMEAs show intense absorption in the visible and near-infrared (NIR) region (650–950 nm) for enhancing the light-harvesting ability [16,17,18]. Compared to NFSMEAs, the development of wide-bandgap (WBG) polymer donors is still limited; only a few donor–acceptor (D–A)-type WBG polymer donors have been reported, such as PM6 and D18 [19,20,21]. Therefore, it is imperative to design novel WBG polymer donors, which absorb the solar energy in the wavelength range of 300–650 nm to further improve the OPV’s performances [22,23,24,25]. Simultaneously, polymers can also be a carrier transport layer in perovskite solar cells [26,27]. Specially, the polymer donors of organic solar cells are often used as the hole transport layers in the perovskite solar cells due to their extraordinary p-type characteristics.

The ideal WBG polymer donors can be synthesized following the donor–acceptor strategy, where benzodithiophene (BDT) [28,29,30] and its derivatives are usually used as the donor units, and some electron-deficient moieties, such as quinoxaline [31,32,33], benzothiadiazole, benzotriazole, and imide, are used as the acceptor co-units [34,35,36]. In addition to these acceptor units, quinoxaline-based polymers have some characteristics that are useful for constructing WBG polymer donors: (1) quinoxaline derivatives possess strong electron-withdrawing properties that can adjust the energy levels; (2) the quinoxaline moiety can be easily modified by introducing sidechains and other functional groups to modulate the physical, chemical, and electronic properties; and (3) quinoxaline has a rigid and large planar structure that favors close π-π stacking. Li et al. [12], reported a low cost quinoxaline-based D–A-type polymer donor, PTQ10, that contains an alkoxy-substituted difluoro quinoxaline acceptor unit and thiophene donor unit on the backbone; when blended with IDIC, a PCE value of 12.7% was achieved for PSCs. Recently, Hou and coworkers reported a WBG polymer donor, PBQx-TCl, based on the thiophene-fused quinoxaline acceptor unit DTQx; the device based on PBQx-TCl:BTP-eC9 exhibited a PCE of 16.0% [14]. Therefore, quinoxaline is a promising building block for constructing high-performance WBG polymer donors in OPVs.

Herein, we design and synthesize a new dibenzo-fused quinoxalineimide (BPQI) building block, which has a rigid and planar structure. By using BDT as the copolymerization unit with BPQI, two new polymer donors, P(BPQI-BDT) and P(BPQI-BDTT), were synthesized. Both polymer donors possess wide bandgaps and deep highest occupied molecular orbital (HOMO) levels, due to the strong electron-withdrawing ability of the BPQI moiety, and have a good compatibility with the Y6 acceptor. As a result, the optimized P(BPQI-BDTT):Y6 device delivers a higher PCE of 6.31% with a *J*_SC_ of 17.09 mA cm^−2^, a *V*_OC_ of 0.82 V, and an FF of 44.78%, compared with the P(BPQI-BDT):Y6 system (PCE of 1.48% with a *J*_SC_ of 6.23 mA cm^−2^, a *V*_OC_ of 0.68 V, and an FF of 34.61%).

## 2. Results and Discussion

### 2.1. Materials Synthesis and Characterization

**Scheme 1:**
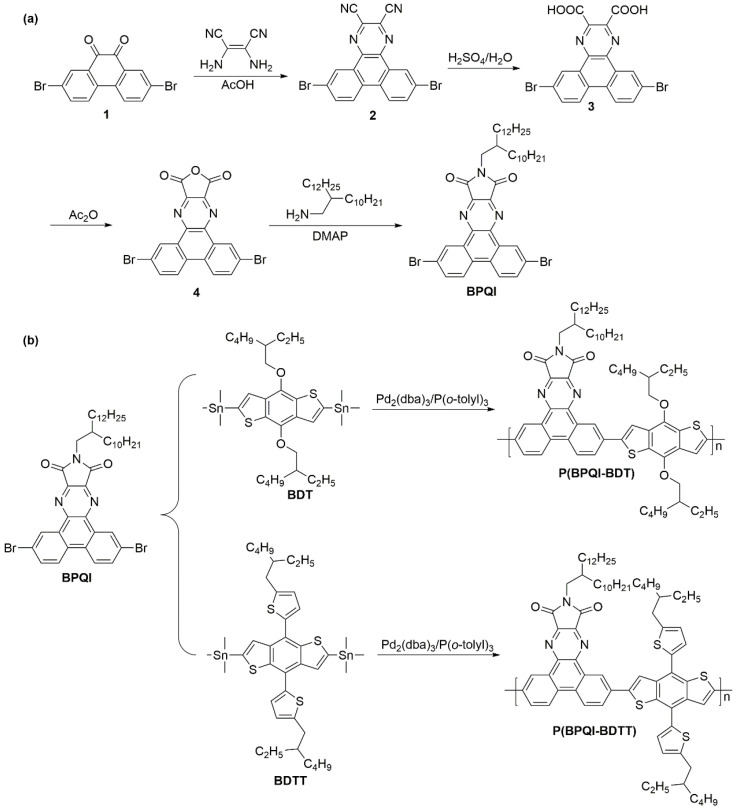
Synthetic route to monomer BPQI (**a**,**b**) and corresponding polymers P(BPQI-BDT) and P(BPQI-BDTT).

The synthetic routes to the monomer BPQI and polymers are displayed in Scheme 1. The detailed synthetic procedures are shown in the Supplementary Materials (Figures S1 and S2). Both polymers P(BPQI-BDT) and P(BPQI-BDTT) are obtained by polymerization of BPQI and the co-monomer BDT derivative. The resulting polymers were purified by Soxhlet extraction with methanol, acetone, hexane, and dichloromethane to remove the catalyst and low molecular weight fractions. The final chloroform fractions were collected and used to test the device’s performance. The numerical average molecular weights (*M*_n_s) and polydispersity indices (PDIs) of P(BPQI-BDT) and P(BPQI-BDTT) were determined by using high-temperature gel permeation chromatography (HT-GPC) at 150 °C with 1,2,4-trichlorobenzene as the eluent. The GPC results are summarized in Table 1: P(BPQI-BDT) has an *M*_n_ of 24 kDa with a PDI of 2.2, and P(BPQI-BDTT) shows an *M*_n_ of 37 kDa with a PDI of 2.0. The thermal properties of P(BPQI-BDT) and P(BPQI-BDTT) were characterized using thermogravimetric analyses (TGA) and differential scanning calorimetry (DSC). Both polymers exhibit excellent thermal stability (5% weight loss) over 300 °C, which indicates good thermal stability for PSC fabrication. Based on the DSC thermograms, no obvious exotherm or endotherm peaks were observed for both polymers P(BPQI-BDT) and P(BPQI-BDTT) in the temperature range from 50 to 300 °C, which likely indicates a low degree of crystallinity in both polymers.

### 2.2. Optical and Electrochemical Properties

**Figure 1:**
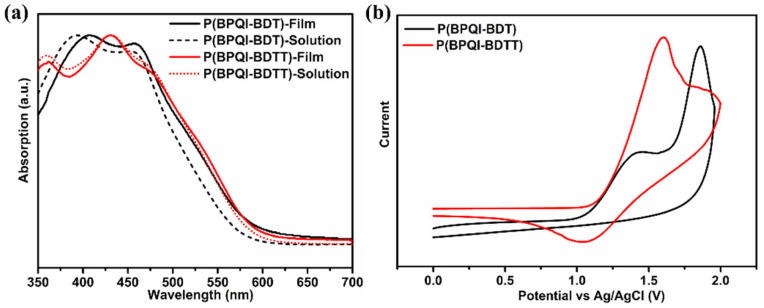
(**a**) Normalized UV-vis absorption spectra of polymers P(BPQI-BDT) and P(BPQI-BDTT) in chloroform solution and as thin films. (**b**) Cyclic voltammogram curves of P(BPQI-BDT) and P(BPQI-BDTT) thin films measured in 0.1 M (*n*-Bu)_4_NPF_6_ acetonitrile solution at a scan rate of 50 mV s^−1^.

The UV-vis absorption spectra of P(BPQI-BDT) and P(BPQI-BDTT) in a chloroform solution and as thin films were shown in Figure 1a, and the corresponding data are summarized in Table 1. In solution, both polymers show two absorption shoulder peaks in the range of 350–500 nm, and the absorption maxima (λ_max_) of P(BPQI-BDT) and P(BPQI-BDTT) in chloroform solution are located at 393 and 432 nm, respectively. In the thin film state, the similar absorption with a slightly red-shifted absorption peak was observed for both polymers, which should be due to close packing in the solid state. The optical bandgaps of P(BPQI-BDT) and P(BPQI-BDTT) are 2.12 and 2.10 eV, respectively, according to the absorption onsets of the polymer thin films.

The electrochemical properties of P(BPQI-BDT) and P(BPQI-BDTT) were characterized by cyclic voltammetry. As shown in Figure 1b, the HOMO energy levels of P(BPQI-BDT) and P(BPQI-BDTT) were calculated from the onset potential of the oxidation peak, which are −5.41 and −5.51 eV (Table 1), respectively. By using the equation of *E*_LUMO_ = (*E*_HOMO_ + *E*_g_) eV, the LUMO energy levels of P(BPQI-BDT) and P(BPQI-BDTT) were found to be −3.29 and −3.41 eV, respectively. The *E*_HOMO_/*E*_LUMO_ of both polymers match well with acceptor Y6, indicating that these two polymers can be used as polymer donor in PSCs.

### 2.3. Theoretical Calculations

**Figure 2:**
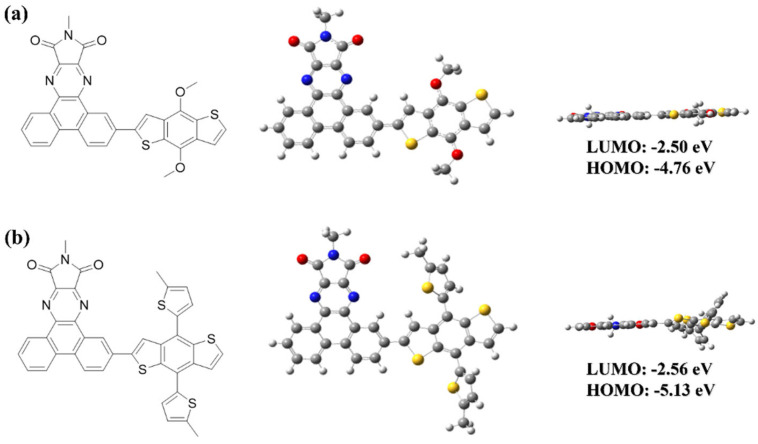
Chemical structures and FMO energy levels of (**a**) P(BPQI-BDT) and (**b**) P(BPQI-BDTT).

Theoretical computation on the backbone geometry of P(BPQI-BDT) and P(BPQI-BDTT) were performed by density functional theory (DFT) using the B3LYP/6-31G level method (Figure 2). The long side chains on the BPQI and BDT were replaced by a methyl group for simplifying the computations, respectively. The DFT-calculated HOMO/LUMO are −4.76/−2.50 and −5.13/−2.56 eV for P(BPQI-BDT) and P(BPQI-BDTT), respectively. The trend of the HOMO/LUMO energy level from the theoretical energy levels are in good agreement with the values obtained by CV measurements.

### 2.4. Photovoltaic Properties

**Figure 3:**
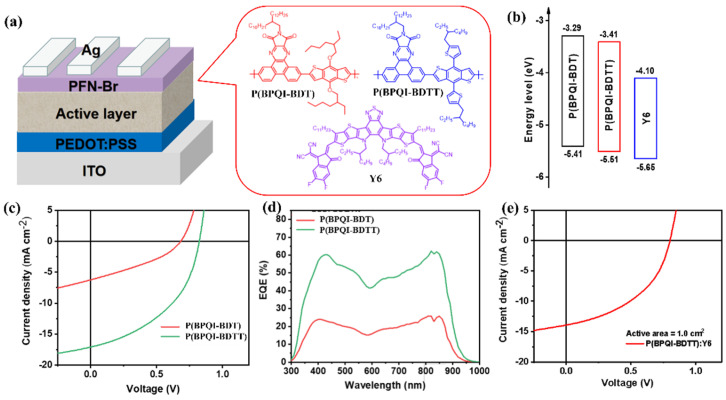
(**a**) Structure of P(BPQI-BDT) or P(BPQI-BDTT) as the polymer donor and Y6 as the acceptor; (**b**) the molecular energy levels of BPQI-BDT, BPQI-BDTT, and Y6; (**c**) vurrent density–applied voltage (*J–V*); (**d**) external quantum efficiency (EQE) curves of P(BPQI-BDT):Y6 or P(BPQI-BDTT):Y6 devices; (**e**) P(BPQI-BDTT):Y6 (*J–V*) curve when active area is 1 cm^2^.

To investigate the photovoltaic properties of P(BPQI-BDT) and P(BPQI-BDTT) in PSCs, the conventional device structure indium tin oxide (ITO)/poly(3,4-ethyl-enedioxythiophene):poly(styrene sulfonic acid) (PEDOT:PSS)/active layer/sliver(Ag)/gold(Au) was fabricated. Y6 was chosen as the acceptor due to its excellent performance in the field of OSCs, and the device architecture is shown in Figure 3a. The molecular energy levels of P(BPQI-BDT) and P(BPQI-BDTT) are shown in Figure 3b. The active layer was deposited by spin-coating the P(BPQI-BDT)/P(BPQI-BDTT):Y6 mixed solution in chloroform at a D/A weight ratio of 1/1, and the solution concentration is 10 mg mL^−1^. The current density–applied voltage (*J–V*) curves are shown in Figure 3c, and the corresponding parameters are summarized in Table 2. As shown in Figure 3c, the optimized P(BPQI-BDTT):Y6-based device delivers a PCE of 6.31% with a *J*_SC_ of 17.09 mA cm^−2^, a *V*_OC_ of 0.82 V, and an FF of 44.78%, whereas the optimized P(BPQI-BDT):Y6-based device displays a low PCE of 1.48% with a *J*_SC_ of 6.23 mA cm^−2^, a *V*_OC_ of 0.68 V, and an FF of 34.61%. The increased *V*_OC_ for the P(BPQI-BDTT)-based PSC is due to the deep-positioned HOMO energy level, since the *V*_OC_ is determined by the HOMO of a donor’s and the LUMO of an acceptor’s energy offset. The external quantum efficiency (EQE) spectra are shown in Figure 3d: the larger *J*_SC_ of the P(BPQI-BDTT):Y6 devices is ascribed to the higher EQE response in the wavelength range from 400 to 850 nm. In contrast, the EQE value of the P(BPQI-BDT):Y6 cells is lower than 25% in this range, resulting in a smaller *J*_SC_. The relationship between *J*_SC_ and the light intensity is revealed in Figure S3a: the α values of the P(BPQI-BDT):Y6 and P(BPQI-BDTT):Y6 devices are 0.92 and 0.974, respectively, which reflects that the P(BPQI-BDTT):Y6 based devices have a smaller bimolecular recombination effect. Figure S3b shows the light-intensity dependence of *V_OC_*. The *n* values of P(BPQI-BDT):Y6 and P(BPQI-BDTT):Y6 are 2.37 and 1.78, respectively. It is shown that the device structure of P(BPQI-BDTT):Y6 produces fewer trap-assisted recombination centers, which are responsible for facilitating charge extraction and improving the PV performance. In order to investigate the device properties of the polymers in more depth, we constructed P(BPQI-BDTT):Y6 devices with a surface area of 1 cm^2^, shown in Figure 3e, and the corresponding parameters are shown in Table 3. The PCE of large device is 5%, with a *V_OC_* of 0.8 V, a *J*_SC_ of 13.92 mA cm^−2^, and an FF of 44.77%. It demonstrated that polymers have the prospect of large-area applications in devices. The commercially polymer PTB7-Th was selected to compare the new polymer P(BPQI-BDTT). The photovoltaic performances of two devices are shown in Figure S4 and Table S1. In Figure S5, we demonstrate the performance of the device in the dark; the variations in the *J*_SC_ and *V*_OC_ are slight, thus showing the stability of the device’s performance in a dark environment.

### 2.5. Polymer Film Morphology

**Figure 4:**
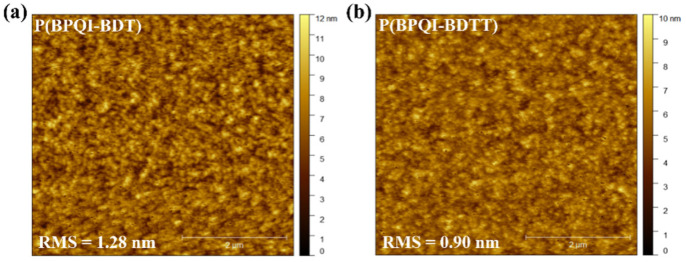
AFM phase images of (**a**) P(BPQI-BDT) and (**b**) P(BPQI-BDTT) blend films, respectively.

Atomic force microscopy (AFM) measurements were taken to study the surface and phase separation morphology of the blend films. As shown in Figure 4, both P(BPQI-BDT):Y6 and P(BPQI-BDTT):Y6 have a relatively smooth surface. The root mean square (RMS) values of roughness for both the P(BPQI-BDT):Y6 and P(BPQI-BDTT):Y6 blend films were 1.28 and 0.9 nm, respectively. It is clear that the P(BPQI-BDT):Y6 blend film has a higher roughness than the P(BPQI-BDTT):Y6 blend, indicating that the P(BPQI-BDT):Y6 blend film enhances the aggregation and phase separation. The P(BPQI-BDTT):Y6 blend has minimal roughness, which is beneficial for charge carrier transportation, charge collection, and less charge recombination, leading to the enhancement of the FF and PCE in OPVs. X-ray diffraction (XRD) was applied to investigate the crystalline properties of these polymers. As shown in Figure S6, the two polymers show wide diffraction and high peaks located at 2*θ* = 37.32° and 2*θ* = 43.62°, which should derive from the two polymers exhibiting similar crystalline surfaces and analogous crystal structures. To further investigate the film morphologies of the polymers, SEM was used to study the surface of the P(BPQI-BDT):Y6 and P(BPQI-BDTT):Y6 films. As shown in Figure S7, it can be seen that these two polymers are irregularly flat films, while P(BPQI-BDTT):Y6 has a more uniform surface morphology, which is favorable for its charge transfer and suppresses charge recombination, effectively improving its device performance.

## 3. Conclusions

In summary, a new dibenzo-fused quinoxalineimide (BPQI) is successfully synthesized as an electron-deficient building block to construct donor–acceptor (D–A)-type polymers, namely P(BPQI-BDT) and P(BPQI-BDTT). Both polymers possess deep HOMO energy levels and wide bandgaps. These two polymers were used as donor materials for polymer solar cells. The PSC based on the P(BPQI-BDTT):Y6 blend showed a higher PCE (6.31%) with a *V*_OC_ of 0.82 V, a *J*_SC_ of 17.09 mA cm^−2^, and an FF of 44.78% as compared to P(BPQI-BDT):Y6 (1.48%) with a *V*_OC_ of 0.68 V, a *J*_SC_ of 6.23 mA cm^−2^, and an FF of 34.61%. The higher PCE is mainly attributed to improvements in the *J*_SC_ and FF. These results demonstrate that BPQI is a promising building block for developing a wide-bandgap polymer donor in OPVs. In addition, the acceptor BPQI can also be applied to other organic opto-electronic devices, such as organic field effect transistors (OFET) and perovskite solar cells (PSCs), due to its large conjugated backbone and electron-deficient characteristics. In future work, a series of quinoxalineimide electron-deficient units will be synthesized, and we believe that quinoxalineimides will become a promising building block for constructing organic semiconductors.

## Figures and Tables

**Table 1:** Molecular Weights and Optical and Electrochemical Properties of Polymers.

Polymer	*M*_n_ (kDa) *^a^*	PDI *^a^*	λ_onset_^film^ (nm) *^b^*	*E*_g_^opt^ (eV) *^c^*	HOMO (eV) *^d^*	LUMO (eV) *^e^*
P(BPQI-BDT)	24	2.2	584	2.12	−5.41	−3.29
P(BPQI-BDTT)	37	2.0	588	2.10	−5.51	−3.41

*^a^* Measured from GPC versus polystyrene standard; trichlorobenzene as the eluent at 150 °C. *^b^* Absorption spectra of the pristine film from chloroform solution. *^c^* Optical bandgap estimated from the absorption onset of the as-cast polymer film using the equation: *E*_g_^opt^ = 1240/λ_onset_ (eV). *^d^ E*_HOMO_ = −e(*E*_ox_^onset^ + 4.80) eV. *^e^ E*_LUMO_ = *E*_HOMO_ + *E*_g_^opt^.

**Table 2:** Photovoltaic parameters of OSCs based on P(BPQI-BDT):Y6’s and P(BPQI-BDTT):Y6’s active layer. *^a^* Average data from 10 independent devices.

Active Layer	*V*_OC_ (V)	*J*_SC_ (mA cm^−2^)	*J*_cal_ (mA cm^−2^)	FF (%)	*PCE_max_/PCE_ave_^a^* (%)
P(BPQI-BDT):Y6	0.68	6.23	6.71	34.61	1.48/1.35 ± 0.10
P(BPQI-BDTT):Y6	0.82	17.09	17.00	44.78	6.31/5.97 ± 0.29

**Table 3:** Photovoltaic parameters of OSCs based on P(BPQI-BDTT):Y6’s active layer with its surface area of 1 cm^2^.

Active Layer	*V*_OC_ (V)	*J*_SC_ (mA cm^−2^)	FF (%)	PCE (%)
P(BPQI-BDTT):Y6	0.80	13.92	44.77	5.00

## Supplementary Materials

The following supporting information can be downloaded at: https://www.mdpi.com/article/10.3390/polym14173590/s1, Figure S1: ^1^H NMR spectrum of compound 5; Figure S2: ^13^C NMR spectrum of compound 5; Figure S3: (a) light intensity dependence of *J*_SC_, (b) light intensity dependence of *V*_OC_; Figure S4: (a) The *J−V* curves and (b) EQE spectra; Figure S5: The *J−V* curves in dark; Figure S6: X-ray diffraction pattern of P(BPQI-BDT) and P(BPQI-BDTT); Figure S7: SEM of film state of P(BPQI-BDT):Y6 and P(BPQI-BDTT):Y6. Table S1: Photovoltaic parameters of OSCs based on P(BPQI-BDTT):Y6 and PTB7-Th:Y6.

## References

[1] Guo X., Zhou N., Lou S.J., Smith J., Tice D.B., Hennek J.W., Ortiz R.P., Navarrete J.T.L., Li S., Strzalka J. (2013). Polymer solar cells with enhanced fill factors. Nat. Photon..

[2] Meng L., Zhang Y., Wan X., Li C., Zhang X., Wang Y., Ke X., Xiao Z., Ding L., Xia R. (2018). Organic and solution-processed tandem solar cells with 17.3% efficiency. Science.

[3] Liu Q., Jiang Y., Jin K., Qin J., Xu J., Li W., Xiong J., Liu J., Xiao Z., Sun K. (2020). 18% Efficiency organic solar cells. Sci. Bull..

[4] Shi Y., Li W., Wang X., Tu L., Li M., Zhao Y., Wang Y., Liu Y. (2022). Isomeric Acceptor–Acceptor Polymers: Enabling Electron Transport with Strikingly Different Semiconducting Properties in n-Channel Organic Thin-Film Transistors. Chem. Mater..

[5] Shi Y., Ma R., Wang X., Liu T., Li Y., Fu S., Yang K., Wang Y., Yu C., Jiao L. (2022). Influence of Fluorine Substitution on the Photovoltaic Performance of Wide Band Gap Polymer Donors for Polymer Solar Cells. ACS Appl. Mater. Interfaces.

[6] Shi Y., Guo H., Huang J., Zhang X., Wu Z., Yang K., Zhang Y., Feng K., Woo H.Y., Ortiz R.P. (2020). Distannylated Bithiophene Imide: Enabling High-Performance n-Type Polymer Semiconductors with an Acceptor-Acceptor Backbone. Angew. Chem. Int. Ed. Engl..

[7] Zhu L., Zhang M., Xu J., Li C., Yan J., Zhou G., Zhong W., Hao T., Song J., Xue X. (2022). Single-junction organic solar cells with over 19% efficiency enabled by a refined double-fibril network morphology. Nat. Mater..

[8] Zou Y., Chen H., Bi X., Xu X., Wang H., Lin M., Ma Z., Zhang M., Li C., Wan X. (2022). Peripheral halogenation engineering controls molecular stacking to enable highly efficient organic solar cells. Energy Environ. Sci..

[9] Wu J., Fan Q., Xiong M., Wang Q., Chen K., Liu H., Gao M., Ye L., Guo X., Fang J. (2020). Carboxylate substituted pyrazine: A simple and low-cost building block for novel wide bandgap polymer donor enables 15.3% efficiency in organic solar cells. Nano Energy.

[10] Zhao J., Li Q., Liu S., Cao Z., Jiao X., Cai Y.-P., Huang F. (2020). Bithieno[3,4-*c*]pyrrole-4,6-dione-Mediated Crystallinity in Large-Bandgap Polymer Donors Directs Charge Transportation and Recombination in Efficient Nonfullerene Polymer Solar Cells. ACS Energy Lett..

[11] Shen Q., He C., Li S., Zuo L., Shi M., Chen H. (2022). Design of Non-fused Ring Acceptors toward High-Performance, Stable, and Low-Cost Organic Photovoltaics. Acc. Mater. Res..

[12] Sun C., Pan F., Bin H., Zhang J., Xue L., Qiu B., Wei Z., Zhang Z.-G., Li Y. (2018). A low cost and high performance polymer donor material for polymer solar cells. Nat. Commun..

[13] Zhang G., Ning H., Chen H., Jiang Q., Jiang J., Han P., Dang L., Xu M., Shao M., He F. (2021). Naphthalenothiophene imide-based polymer exhibiting over 17% efficiency. Joule.

[14] Xu Y., Cui Y., Yao H., Zhang T., Zhang J., Ma L., Wang J., Wei Z., Hou J. (2021). A New Conjugated Polymer that Enables the Integration of Photovoltaic and Light-Emitting Functions in One Device. Adv. Mater..

[15] Zhu C., Meng L., Zhang J., Qin S., Lai W., Qiu B., Yuan J., Wan Y., Huang W., Li Y. (2021). A Quinoxaline-Based D-A Copolymer Donor Achieving 17.62% Efficiency of Organic Solar Cells. Adv. Mater..

[16] Wang Y., Guo H., Ling S., Arrechea-Marcos I., Wang Y., Navarrete J.T.L., Ortiz R.P., Guo X. (2017). Ladder-type Heteroarenes: Up to 15 Rings with Five Imide Groups. Angew. Chem. Int. Ed. Engl..

[17] Luo Z., Ma R., Yu J., Liu H., Liu T., Ni F., Hu J., Zou Y., Zeng A., Su C.-J. (2022). Heteroheptacene-based acceptors with thieno[3,2-b]pyrrole yield high-performance polymer solar cells. Natl. Sci. Rev..

[18] Li T., Wu Y., Zhou J., Li M., Wu J., Hu Q., Jia B., Pan X., Zhang M., Tang Z. (2020). Butterfly Effects Arising from Starting Materials in Fused-Ring Electron Acceptors. J. Am. Chem. Soc..

[19] Shi Y., Tang Y., Yang K., Qin M., Wang Y., Sun H., Su M., Lu X., Zhou M., Guo X. (2019). Thiazolothienyl imide-based wide bandgap copolymers for efficient polymer solar cells. J. Mater. Chem. C.

[20] An N., Cai Y., Wu H., Tang A., Zhang K., Hao X., Ma Z., Guo Q., Ryu H.S., Woo H.Y. (2020). Solution-Processed Organic Solar Cells with High Open-Circuit Voltage of 1.3 V and Low Non-Radiative Voltage Loss of 0.16 V. Adv. Mater..

[21] An Q., Wang J., Ma X., Gao J., Hu Z., Liu B., Sun H., Guo X., Zhang X., Zhang F. (2020). Two compatible polymer donors contribute synergistically for ternary organic solar cells with 17.53% efficiency. Energy Environ. Sci..

[22] Guo X., Fan Q., Wu J., Li G., Peng Z., Su W., Lin J., Hou L., Qin Y., Ade H. (2021). Optimized Active Layer Morphologies via Ternary Copolymerization of Polymer Donors for 17.6% Efficiency Organic Solar Cells with Enhanced Fill Factor. Angew. Chem. Int. Ed. Engl..

[23] Ha J.-W., Kim H.S., Song C.E., Park H.J., Hwang D.-H. (2020). Thienoquinolinone as a new building block for wide bandgap semiconducting polymer donors for organic solar cells. J. Mater. Chem. C.

[24] Luo Y., Luo Y., Huang X., Liu S., Cao Z., Guo L., Li Q., Cai Y., Wang Y. (2021). A New Ester-Substituted Quinoxaline-Based Narrow Bandgap Polymer Donor for Organic Solar Cells. Macromol. Rapid Commun..

[25] Xie Q., Liu Y., Liao X., Cui Y., Huang S., Hu L., He Q., Chen L., Chen Y. (2020). Isomeric Effect of Wide Bandgap Polymer Donors with High Crystallinity to Achieve Efficient Polymer Solar Cells. Macromol. Rapid Commun..

[26] Khadka D.B., Shirai Y., Yanagida M., Noda T., Miyano K. (2018). Tailoring the Open-Circuit Voltage Deficit of Wide-Band-Gap Perovskite Solar Cells Using Alkyl Chain-Substituted Fullerene Derivatives. ACS Appl. Mater. Interfaces.

[27] Nakanishi R., Nogimura A., Eguchi R., Kanai K. (2014). Electronic structure of fullerene derivatives in organic photovoltaics. Org. Electron..

[28] Zhang Y., Liu D., Lau T., Zhan L., Shen D., Fong P.W.K., Yan C., Zhang S., Lu X., Lee C.-S. (2020). A Novel Wide-Bandgap Polymer with Deep Ionization Potential Enables Exceeding 16% Efficiency in Ternary Nonfullerene Polymer Solar Cells. Adv. Funct. Mater..

[29] Zhang T., Zeng G., Ye F., Zhao X., Yang X. (2018). Efficient Non-Fullerene Organic Photovoltaic Modules Incorporating As-Cast and Thickness-Insensitive Photoactive Layers. Adv. Energy Mater..

[30] Zhang W., Huang J., Xu J., Han M., Su D., Wu N., Zhang C., Xu A., Zhan C. (2020). Phthalimide Polymer Donor Guests Enable over 17% Efficient Organic Solar Cells via Parallel-Like Ternary and Quaternary Strategies. Adv. Energy Mater..

[31] Keshtov M.L., Khokhlov A.R., Godovsky D.Y., Ostapov I.E., Alekseev V.G., Xie Z., Chayal G., Sharma G.D. (2022). Novel Pyrrolo [3,4-b] Dithieno [3, 2-f:2″,3″-h] Quinoxaline-8,10 (9H)-Dione Based Wide Bandgap Conjugated Copolymers for Bulk Heterojunction Polymer Solar Cells. Macromol. Rapid Commun..

[32] Busireddy M.R., Chen T.-W., Huang S.-C., Nie H., Su Y.-J., Chuang C.-T., Kuo P.-J., Chen J.-T., Hsu C.-S. (2022). Fine Tuning Alkyl Substituents on Dithienoquinoxaline-Based Wide-Bandgap Polymer Donors for Organic Photovoltaics. ACS Appl. Mater. Interfaces.

[33] Sun C., Zhu C., Meng L., Li Y. (2022). Quinoxaline-Based D–A Copolymers for the Applications as Polymer Donor and Hole Transport Material in Polymer/Perovskite Solar Cells. Adv. Mater..

[34] Yong-qiang S., Wang Y., Guo X. (2019). Recent Progress of Imide-Functionalized N-Type Polymer Semiconductors. Acta. Polym. Sin..

[35] Shi Y., Guo H., Qin M., Wang Y., Zhao J., Sun H., Wang H., Wang Y., Zhou X., Facchetti A. (2018). Imide-Functionalized Thiazole-Based Polymer Semiconductors: Synthesis, Structure–Property Correlations, Charge Carrier Polarity, and Thin-Film Transistor Performance. Chem. Mater..

[36] Shi Y., Guo H., Qin M., Zhao J., Wang Y., Wang H., Wang Y., Facchetti A., Lu X., Guo X. (2018). Thiazole Imide-Based All-Acceptor Homopolymer: Achieving High-Performance Unipolar Electron Transport in Organic Thin-Film Transistors. Adv. Mater..

