# Supplementary material for: A New Dibenzoquinoxalineimide-Based Wide-Bandgap Polymer Donor for Polymer Solar Cells
Source: Polymers (Basel). 2022 Aug 30;14(17):3590. doi: 10.3390/polym14173590 (PMC9460915; doi:10.3390/polym14173590)
Supplement: Supplementary file 1 [file polymers-14-03590-s001.zip › polymers-1858937-supplementary.pdf]

polymers

Supplementary Material: A New Dibenzoquinoxalineimide-Based Wide Bandgap Polymer Donor for Polymer Solar Cells

Xin Wang, Zongtao Wang, Mingwei Li, Lijun Tu, Ke Wang, Dengping Xiao, Qiang Guo, Ming Zhou, Xianwen Wei, Yongqiang Shi and Erjun Zhou

Materials and Methods

Chemicals and reagents were commercial available and used without further purification. Anhydrous tetrahydrofuran and toluene were distilled from Na/benzophenone, and anhydrous dichloromethane and acetonitrile were distilled from CaH_2 under argon. 2,7-Dibromophenanthrene-9.10-dione and Diaminomaleonitrile were purchased from Bide pharmatech. (Shanghai, China). UV-Vis absorption spectra were recorded on a Shimadzu UV-3600 UV-VIS-NIR spectrophotometer. Cyclic voltammetry measurements of polymer films were performed under argon atmosphere using a CHI760E voltammetric analyzer with 0.1 M tetra-n-butylammonium hexafluorophosphate in acetonitrile as the supporting electrolyte. ^{1}H and ^{13}C nuclear magnetic resonance spectra were tested on a Bruker Ascend 400 MHz spectrometer.A platinum disk working electrode, a platinum wire counter electrode, and a silver wire reference electrode were employed, and the ferrocene/ferrocenium (Fc/Fc^{+}) was used as the internal reference for all measurements. The scan rate was 50 mV/s. Polymer film was drop-coated from chloroform solutions on a Pt working electrode (2 mm in diameter). The supporting electrolyte solution was thoroughly purged with argon before all CV measurements. AFM measurements of polymer films were performed by using a Dimension Icon Scanning Probe Microscope (Asylum Research, MFP-3D-Stand Alone) in tapping mode.

Synthesis of Monomer.

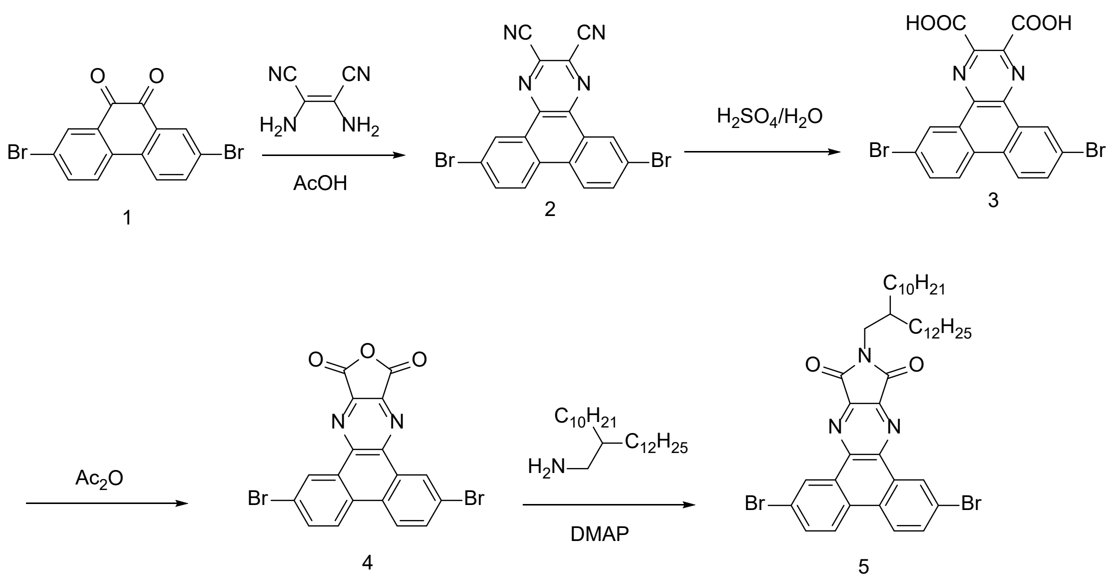

Scheme S1. Synthetic route to the BPQI.

Synthesis of compound 2.

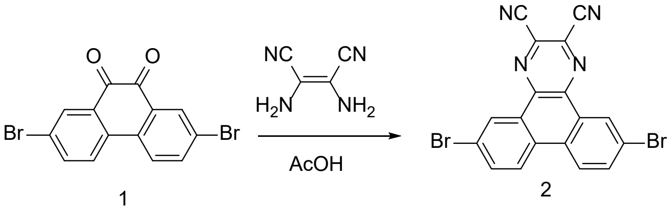

To a 100-mL round-bottom flask,containing a stir bar,and under argon atmosphere, was added 2,7-Dibromophenanthrene (1g, 2.7 mmol) , Diaminomaleonitrile (321 mg, 2.97 mmol) , and 60mL acetic acid. The flask equipped with a condenser and the mixture was warmed to reflux (120-130℃), and stirred for 3 hours. The reation mixture was cooled to room temperature, then poured into water and filtered. The resulting solid was dispersed in water , filtered, washed with plenty water and cold ethanol. The resulting earth-yellow solid was air-dried 1 minute, placed under vacuum.

Synthesis of compound 3.

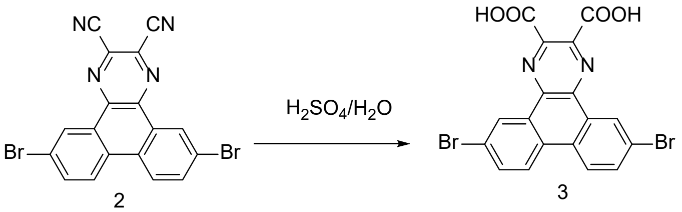

To a 100-mL round-bottom flask,containing a stir bar,and under argon atmosphere, compound **2** (1 g, 2.28mmol) was added, 50mL concentrated sulfuric acid, and 50mL water. The flask was equipped with a condenser and the mixture was warmed to reflux (120-130℃), and strirred for 20 hours. The reaction was cooled to room temperature, poured into 500mL ice water. The yellow solid was dispersed in water, filtered, washed with plenty water. The resulting yellow solid was dried under vacuum.

Synthesis of compound 4.

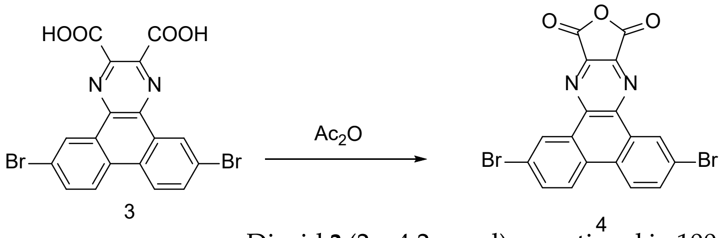

Diacid **3** (2g, 4.2mmol) was stirred in 100mL of acetic anhydride at reflux for 6h. Upon cooling to 0℃, the solid was collected by filtration, washed with 50 mL hexane, and dried in vacuum at 120℃ overnight. The resulting yellow solid (1.96g, 98% yield) were used without further purification.

Synthesis of compound 5.

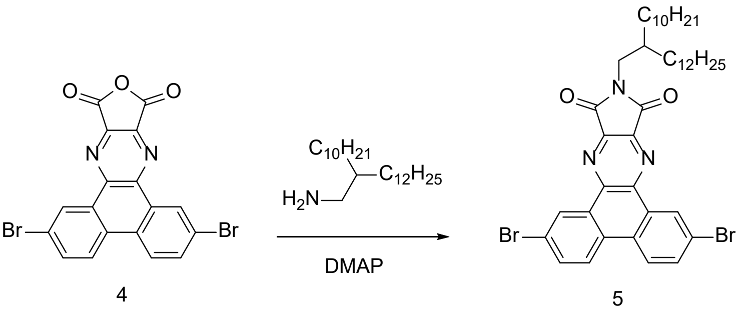

To a 100-mL two neck round-bottom flask,,containing a stir bar, and under argon atmosphere, compound 4 was added, DMAP (122mg, 0.72mmol), 2-decyltetradecan-1-amine, and 50mL anhydrous methylene chloride. The flask equipped with a condenser and the mixture was warmed to reflux (50-60°C), and strirred for 10 hours. The solvent removed by evaporation, acetic anhydride was added. The mixture was warmed to reflux (135-145°C), and strirred for 10 hours. The solvent removed under reduced pressure, the residue was treated with water (50 mL), extracted with CH_2Cl_2 (50 mL×2).The combined organic layers were washed with water and brine successively, dried over anhydrous magnesium sulfate. Evaporation of the solvent under rotary evaporator, the crude product was purified by column chromatography using hexane/methylene chloride (1:1) as an eluent to afford compound BQ-EDOT as an yellow solid. ^{1}H NMR (400MHz, CDCl3) δ : 9.53 (d, J= 3.8 Hz, 2H), 8.13 (d, J = 3.2 Hz, 2H), 7.99 (d, J = 3.2 Hz,2H), 3.83 (d, J= 1.5 Hz, 2H), 1.39-1.25(m, 40H), 0.88 (m, 4H). ^{13}C NMR (126 MHz, CDCl3) δ: 164.43 , 144.91, 143.18, 134.65, 130.26, 130.14, 129.43, 124.44, 123.33, 43.23, 37.25, 31.93, 31.53, 29.99, 29.70, 29.66, 29.36, 29.29, 26.29, 22.70, 14.14.

Synthesis of Polymers.

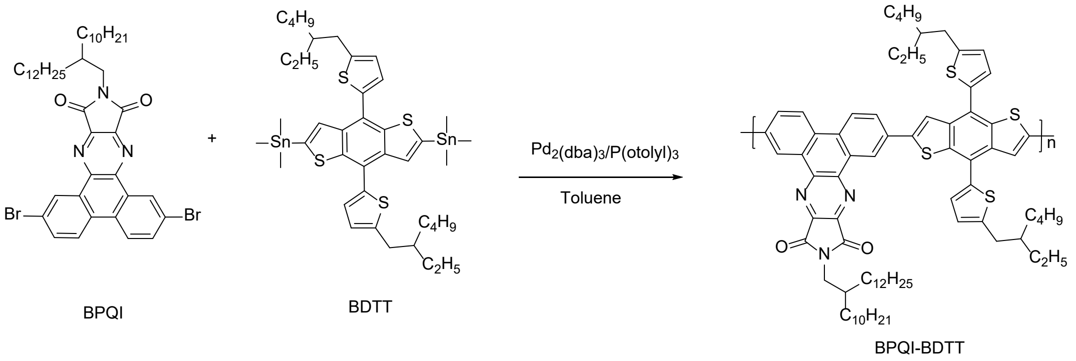

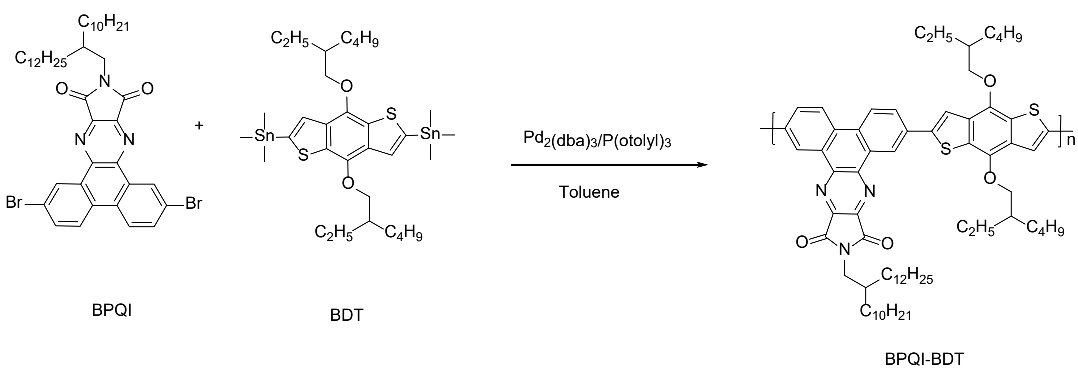

An glass tube charged with two monomer (0.1mmol each), tris(dibenzylideneacetone)dipalladium (0) ($Pd_2(dba)_3$), and tris(*o*-tolyl)3) at 1:8 molar ratio, Pd (0) was 0.03-0.05equiv. The tube under argon atmosphere, the anhydrous toluene 3 mL was added. The tube was sealed and then stirred at 120 °C for 20 hours. Then, 0.1 mL 2-(tributylstanny)thiophene was added and the reaction mixture was stirred at 120°C for 0.5 h. Finally, 0.2 mL 2-bromothiophene was added and the reaction mixture was stirred at 120°C for another 0.5 h. After cooling to room temperature, 2 mL HCl was added to the mixture. The mixture was slowly dripped into 100mL methanol. The precipitate was filter by gravity. The solid was transferred to a Soxhlet thimble. After drying, the crude product

was subjected to sequential Soxhlet extraction with the solvent sequence of methanol, acetone, hexane, and chloroform. The chloroform fraction was concentrated by removing most of solvent under reduced pressure and then precipitated into methanol. The solid was collected by filtration and dried in vacuum to afford the polymer as a deep colored solid .

P(BPQI-BDT): Anal. Calcd. for $C_{68}H_{93}N_3O_4S_2$ (%) : C, 75.58; H, 8.67; N, 3.89; S, 5.93. Found (%): C, 75.01; H, 8.48; N, 3.98; S, 5.85.

P(BPQI-BDT): Anal. Calcd. for $C_{76}H_{97}N_3O_2S_4$ (%) : C, 75.26; H, 8.06; N, 3.46; S, 10.57. Found (%): C, 75.81; H, 8.67; N, 3.66; S, 11.01.

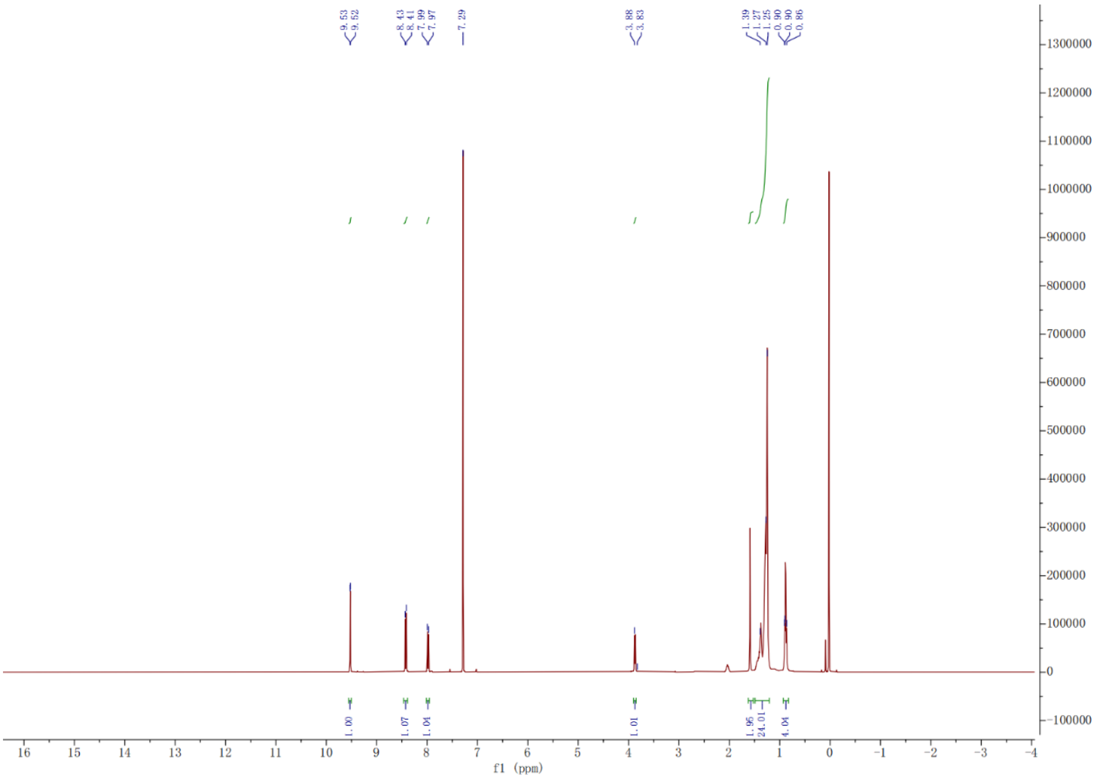

Figure S1. ^{1}H NMR spectrum of compound 5 (400 M,r.t., in $CDCl_3$).

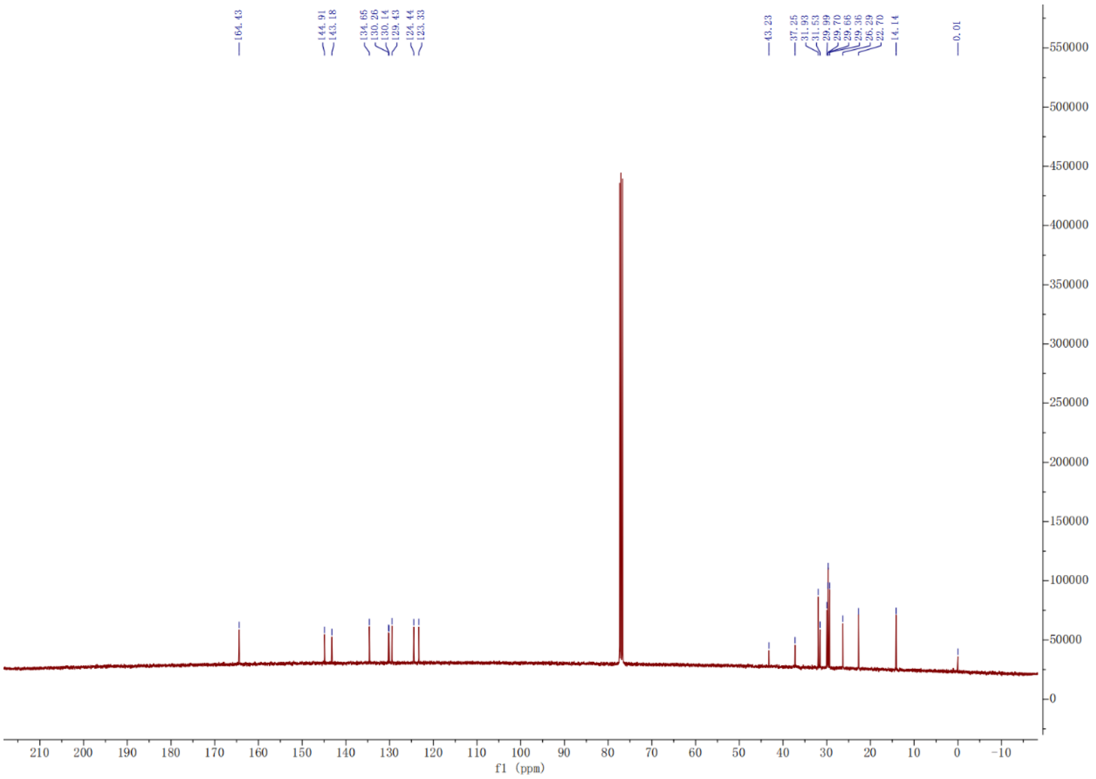

Figure S2. ^{13}C NMR spectrum of compound 5 (400 M,r.t., in $CDCl_3$).

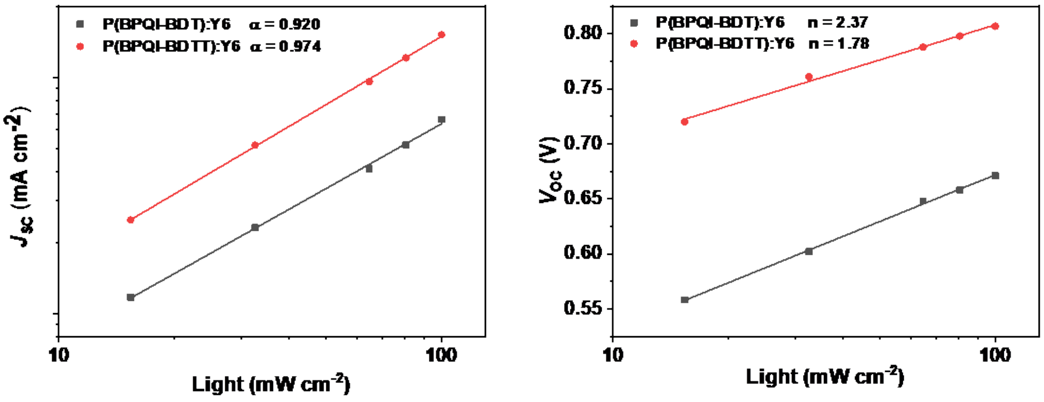

Figure S3. (a) light intensity dependence of J_{SC}; (b) light intensity dependence of V_{OC}.

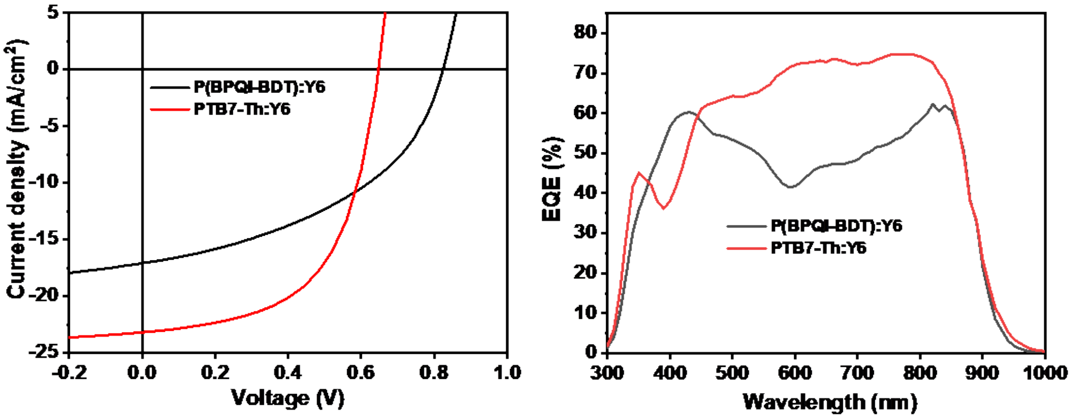

Figure S4. (a) The *J–V* curves and (b) EQE spectra.

Table S1. Photovoltaic parameters of OSCs based on P(BPQI-BDTT):Y6 and PTB7-Th:Y6.

Active Layer	V_{OC} (V)	J_{SC} (mA cm^{-2})	J_{cal} (mA cm^{-2})	FF (%)	PCE (%)
P(BPQI-BDTT):Y6	0.82	17.09	17.00	44.78	6.31
PTB7-Th:Y6	0.65	23.18	22.03	57.31	8.59

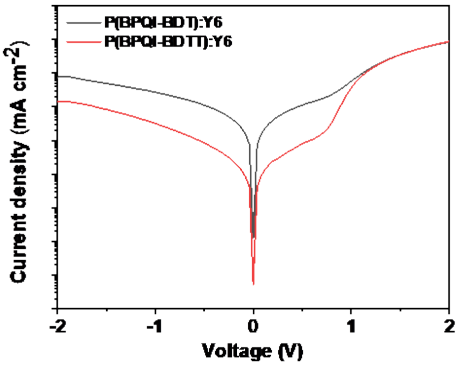

Figure S5. The *J–V* curves in dark.

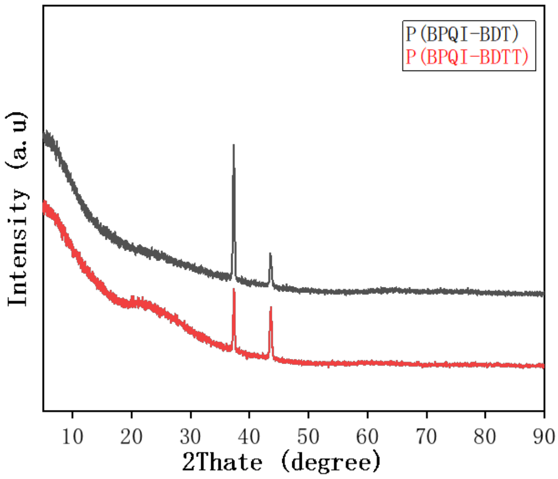

Figure S6. X-ray diffraction pattern of P(BPQI-BDT) and P(BPQI-BDTT).

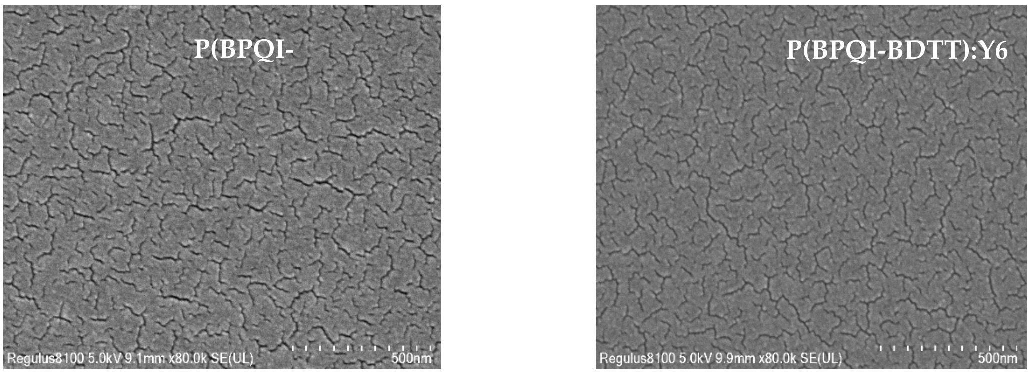

Figure S7. SEM of film state of P(BPQI-BDT):Y6 and P(BPQI-BDTT):Y6
